# Structure and signaling at hydroid polyp-stolon junctions, revisited

**DOI:** 10.1242/bio.012187

**Published:** 2015-07-31

**Authors:** Katherine L. Harmata, Emily L. Somova, Austin P. Parrin, Lori S. Bross, Sally L. Glockling, Neil W. Blackstone

**Affiliations:** Department of Biological Sciences, Northern Illinois University, DeKalb, IL 60115, USA

**Keywords:** Cnidarians, Metabolism, Mitochondria, Reactive oxygen species

## Abstract

The gastrovascular system of colonial hydroids is central to homeostasis, yet its functional biology remains poorly understood. A probe (2′,7′-dichlorodihydrofluorescein diacetate) for reactive oxygen species (ROS) identified fluorescent objects at polyp-stolon junctions that emit high levels of ROS. A nuclear probe (Hoechst 33342) does not co-localize with these objects, while a mitochondrial probe (rhodamine 123) does. We interpret these objects as mitochondrion-rich cells. Confocal microscopy showed that this fluorescence is situated in large columnar cells. Treatment with an uncoupler (2,4-dinitrophenol) diminished the ROS levels of these cells relative to background fluorescence, as did removing the stolons connecting to a polyp-stolon junction. These observations support the hypothesis that the ROS emanate from mitochondrion-rich cells, which function by pulling open a valve at the base of the polyp. The open valve allows gastrovascular fluid from the polyp to enter the stolons and vice versa. The uncoupler shifts the mitochondrial redox state in the direction of oxidation, lowering ROS levels. By removing the stolons, the valve is not pulled open, metabolic demand is lowered, and the mitochondrion-rich cells slowly regress. Transmission electron microscopy identified mitochondrion-rich cells adjacent to a thick layer of mesoglea at polyp-stolon junctions. The myonemes of these myoepithelial cells extend from the thickened mesoglea to the rigid perisarc on the outside of the colony. The perisarc thus anchors the myoepithelial cells and allows them to pull against the mesoglea and open the lumen of the polyp-stolon junction, while relaxation of these cells closes the lumen.

## INTRODUCTION

Increasingly, the cnidarian gastrovascular system is regarded as an integrator of information that maintains homeostasis, particularly in colonial forms (Schierwater et al., 1992; Buss, 2001; Blackstone et al., 2005; Parrin et al., 2010). For this gastrovascular system, available data suggest that cilia-driven flow is plesiomorphic, while flow driven by myoepithelial cells is derived (Harmata et al., 2013). Colonial hydroids represent one of the groups that exhibit the derived condition, yet it remains unclear how myoepithelial cells actually produce the complex patterns of gastrovascular flow in a colony.

**Fig. 1.**
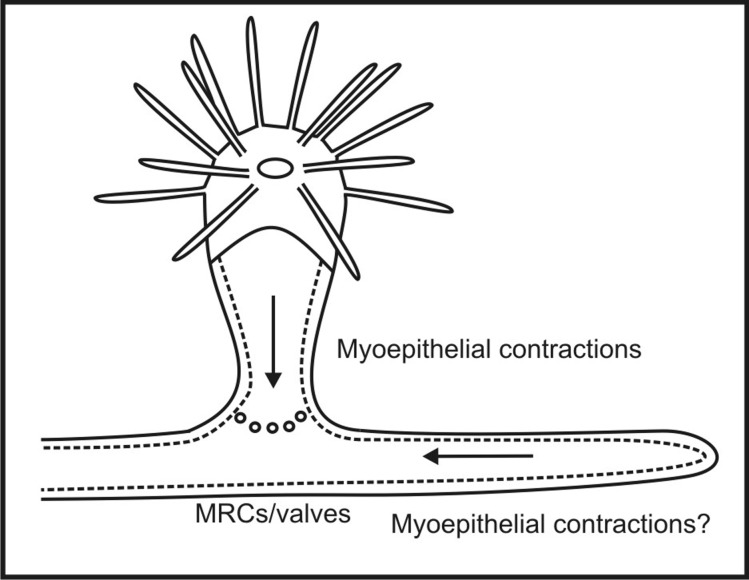
**A hypothesis for how gastrovascular flow is regulated in colonial hydroids.** At the polyp-stolon junctions there are mitochondrion-rich myoepithelial cells (MRCs) and valve-like structures; these valves in part regulate the flow (Blackstone et al., 2004a,b). Additional regulation may be provided by myoepithelial contractions of the polyps and possibly the stolons.

Recently discovered mitochondrion-rich myoepithelial cells at the junction of polyps and stolons may have a central role in regulating the patterns of gastrovascular flow in a colony (Fig. 1). These cells may operate a valve-like structure at the polyp-stolon junction (Blackstone et al., 2004a,b). The existence of such valves explains a variety of observations, including the delay between feeding and the circulation of food-containing gastrovascular fluid from the polyps (Dudgeon et al., 1999). Further, the large numbers of mitochondria in these cells suggest that operating these valves is metabolically demanding. In animal cells, biogenesis of mitochondria is thought to be regulated by PGC-1α (peroxisome proliferator-activated receptor-γ coactivator-1α). PGC-1α is a potent metabolic sensor and regulator (Wu et al., 1999; Cunningham et al., 2007; Arany et al., 2008). In response to a high level of metabolic demand indicated by a low ATP/ADP ratio and low to moderate reactive oxygen species (ROS), PGC-1α will initiate mitochondrial biogenesis. This suggests that any metazoan cell that is subject to intense metabolic demand will become mitochondrion-rich. Indeed, such cells are found in human organs with high metabolism (e.g. brain, heart, and kidney). Salt glands of marine birds and the gills of some fish also exhibit mitochondrion-rich cells (Chance et al., 1964; Evans et al., 2005; Hiroi et al., 2005).

Mitochondrion-rich cells have been identified using spectrofluorometry and fluorescent microscopy. Using living colonies, early studies identified and quantified the blue light emissions subsequent to excitation in the ultraviolet (Blackstone and Buss, 1992, 1993; Blackstone, 1998a). These emissions could be diminished using the uncoupler 2,4-dinitrophenol (DNP)(Blackstone and Buss, 1992). The effects of DNP were important because hydroid colonies contain chitin as a structural element. When excited in the ultraviolet, chitin also fluoresces strongly in the blue range. The effects of DNP suggest that the fluorescence was due to NAD(P)H. Uncouplers such as DNP diminish the transmembrane proton gradient of mitochondria, shifting the redox state in the direction of oxidation. Since NAD(P)^+^ does not fluoresce, fluorescence of mitochondrial NAD(P)H is thus diminished. At the same time, no effect of uncouplers on fluorescence of chitin is expected or observed.

Fluorescence of NAD(P)H has been and continues to be widely used to assay metabolic state (Chance and Thorell, 1959; Chance, 1991; Dumas et al., 2009; Quinn et al., 2013). After a number of years of carrying out such measures using spectrofluorometry, colonies were examined with fluorescent microscopy to better localize the source of the NAD(P)H. Surprisingly, discrete fluorescent objects several micrometers in diameter were detected at the polyp-stolon junctions. Tentatively, these were interpreted as extensions of the myoepithelial cells in polyps (Blackstone, 1998b). This fluorescence could still be diminished by uncouplers, so it was again attributed to NAD(P)H.

NAD(P)H and FAD emissions from the upper part of the polyp were analyzed using flash-frozen colonies and a time-sharing fluorometer (Chance et al., 1971). This technique allowed visualizing the upper polyps, which lie outside the focal plane of microscopy. No strong metabolic emissions comparable to those at polyp-stolon junctions were detected (N.W.B and Britton Chance, unpublished). This result cast doubt on the hypothesis that the NAD(P)H emissions from the polyp-stolon junctions were related to the myoepithelial cells of the polyps.

An examination of the ultrastructure of hydroid polyps, stolons, and polyp-stolon junctions using transmission electron microscopy (TEM) provided further insight. Mitochondrion-rich cells were only found in a narrow band at polyp-stolon junctions on the ectodermal side of an area in which the mesoglea was thickened. At the same time, *in vivo* examination of the fluorescent objects of Blackstone (1998b) demonstrated that the native fluorescence of NAD(P)H co-localizes with ROS-related emissions from 2′,7′-dichlorodihydrofluorescein diacetate (H_2_DCFDA) (Blackstone, 2001, 2003), and emissions from rhodamine 123, a mitochondrial probe (Blackstone et al., 2004a,b). The nature of these cells and the functional biology was thus re-interpreted in subsequent publications.

**Abbreviations**

DMSO: Dimethyl sulfoxide
DNP: 2,4-dinitrophenol
MRCs: mitochondrion-rich myoepithelial cells
PGC-1α: peroxisome proliferator-activated receptor-γ coactivator-1α
ROS: reactive oxygen species
TEM: transmission electron microscopy
H_2_DCFDA: 2′,7′-dichlorodihydrofluorescein diacetate

We take this opportunity to re-evaluate the structure and signaling of polyp-stolon junctions in the hydroid gastrovascular system. The evidence presented continues to suggest that the valves are structurally complex and functionally important.

## RESULTS

### Elucidation of the structure of polyp-stolon junctions

The probe H_2_DCFDA is commonly used to visualize ROS in cnidarians (Lesser, 1996; Jantzen et al., 1998; Blackstone, 2001; Franklin et al., 2004; Chen et al., 2012) as well as in other organisms (Nishikawa et al., 2000; Pei et al., 2000). Outside of a cell this probe is non-fluorescent. When it is taken up by a living cell, esterases can remove the acetate groups and if it is oxidized (typically by peroxide), it is converted to fluorescent DCF. H_2_DCFDA thus serves as a semi-quantitative measure of ROS in living cells. Confounding effects of native fluorescence can be examined with negative controls at the same emission (green) and excitation wavelengths (blue) and otherwise identical experimental conditions. Here we use this probe and others to examine polyp-stolon junctions in *Podocoryna carnea* Sars, and *Hydractinia symbiolongicarpus* Buss & Yund. Similar results for both species were obtained, but only the results for colonies of *P. carnea* are presented*.*

**Fig. 2.**
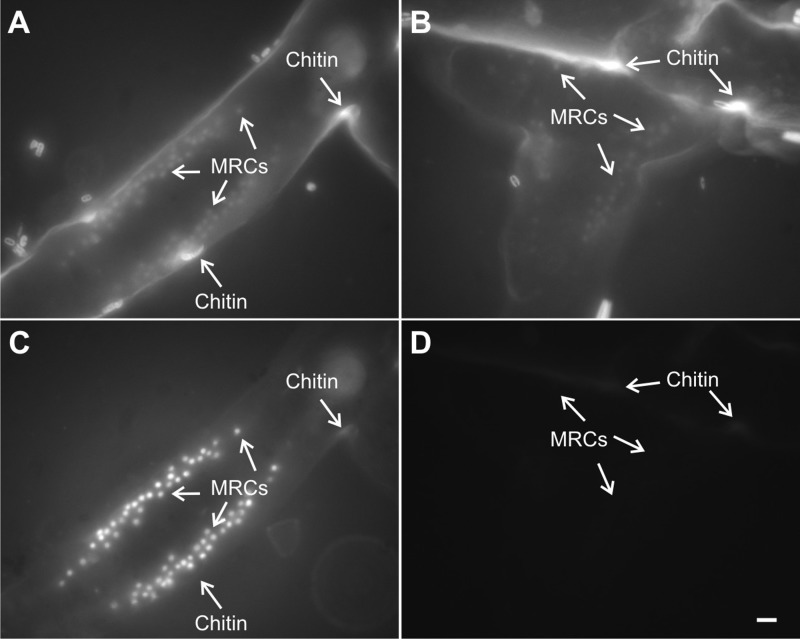
**Micrographs of fluorescent emissions from polyp-stolon junctions in living colonies of *Podocoryna carnea* treated with H_2_DCFDA.** A polyp-stolon junction of a colony treated with H_2_DCFDA is shown with ultraviolet excitation, blue emission (A) and blue excitation, green emission (C). A polyp-stolon junction of a colony that was treated with an equivalent amount of DMSO (negative control), is shown with ultraviolet excitation, blue emission (B) and blue excitation, green emission (D). Identical camera settings were used for A and B, and for C and D. MRCs, mitochondrion-rich cells. Scale bar: 10 µm.

Mitochondrion-rich cells are expected to emit large quantities of ROS (Chance et al., 1979). Hence, H_2_DCFDA should be oxidized by these cells in great quantities (Blackstone, 2001; Blackstone et al., 2004a,b). These results (Fig. 2) confirm earlier work (Blackstone, 2001, 2003; Blackstone et al., 2004a,b). With excitement in the ultraviolet and emission in the blue, the chitinous perisarc on the outside of the colony strongly fluoresced. The putative mitochondrion-rich cells also fluoresced. The NAD(P)H and chitin emissions were impossible to distinguish. In colonies that were incubated in H_2_DCFDA, however, the intense emission from DCF required setting the camera to much lower sensitivity. Native fluorescence, whether of FAD or chitin, was virtually undetectable at these settings (negative control, Fig. 2D). The mitochondrion-rich cells nevertheless strongly fluoresced (Fig. 2C). The negative controls showed that native fluorescence, whether of FAD or chitin, did not contribute at all to this fluorescence (compare Fig. 2C to D).

**Fig. 3.**
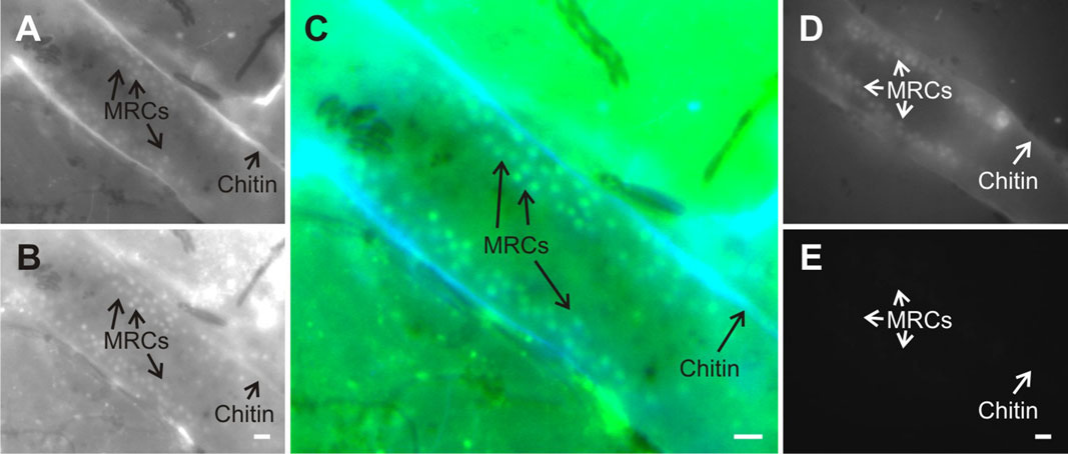
**Micrographs of fluorescent emissions from polyp-stolon junctions in living colonies of *Podocoryna carnea* treated with rhodamine 123.** A polyp-stolon junction of a colony treated with rhodamine 123 (A,B) and of a colony incubated in seawater (negative control) (D,E) as in Fig. 2. A merged, pseudocolored image for A and B is shown (C). With a region of interest confined to the polyp-stolon junction, co-localization (Pearson's correlation, r_p_) of the two emissions is 0.86, which suggests a high degree of co-localization. MRCs, mitochondrion-rich cells. Scale bar: 10 µm.

**Fig. 4.**
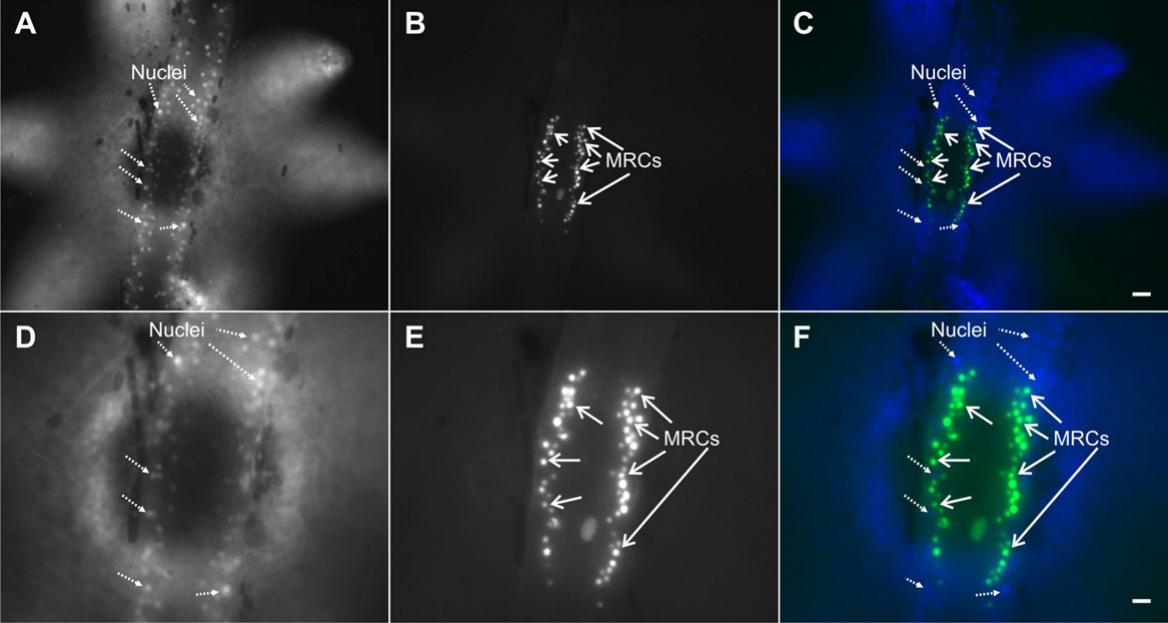
**Micrographs of fluorescent emissions from a polyp-stolon junction in a living colony of *Podocoryna carnea* simultaneously treated with Hoechst 33342 and H_2_DCFDA.** For this colony, the same polyp-stolon junction is shown at two different magnifications with ultraviolet excitation, blue emission, which visualizes nuclei (A,D) and blue excitation, green emission, which visualizes ROS (B,E). Merged, pseudocolored images are also shown (C,F). With a region of interest confined to the polyp-stolon junction, co-localization (Pearson's correlation, r_p_) of the two probes is −0.07 (C) and 0.04 (F), which suggests little or no co-localization. MRCs, mitochondrion-rich cells (exclusive of their nuclei). Scale bars: A-C, 20 µm; D-F, 10 µm.

**Fig. 5.**
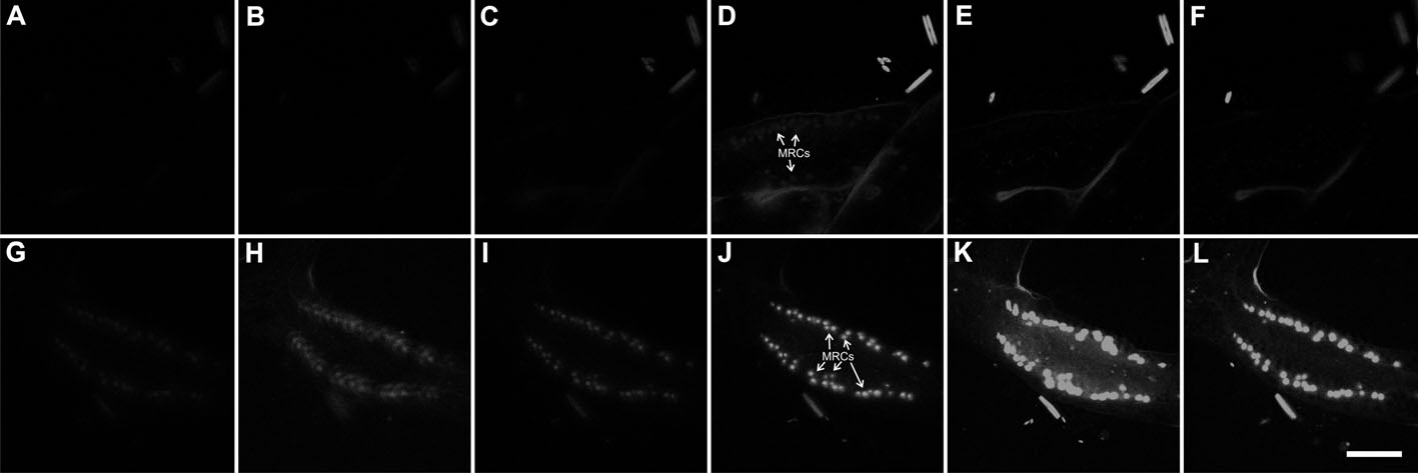
**Confocal sections of a polyp-stolon junction from a living colony of *Podocoryna carnea* treated with H_2_DCFDA.** A negative control (A-F) and a treated colony (G-L) are shown. Starting with the base (A and G), the panels (B-F and H-L) depict every 10th optical section of a polyp-stolon junction. Each section is 0.47 µm thick. MRCs, mitochondrion-rich cells. Scale bar: 50 µm.

Fluorescence of rhodamine 123, a mitochondrial probe (Johnson et al., 1980), also co-localized with NAD(P)H emissions (Fig. 3). On the other hand, Hoechst 33342, a nuclear probe (Dunn et al., 2012), did not co-localize with H_2_DCFDA emissions (Fig. 4). H_2_DCFDA-derived fluorescence was also examined using confocal microscopy, which shows fluorescence primarily localized to columnar cells in the upper part of the poly-stolon junction (Fig. 5). Three-dimensional reconstruction of the image stack shows two crests of mitochondrion-rich cells on both sides of the polyp-stolon junction (data not shown).

**Fig. 6.**
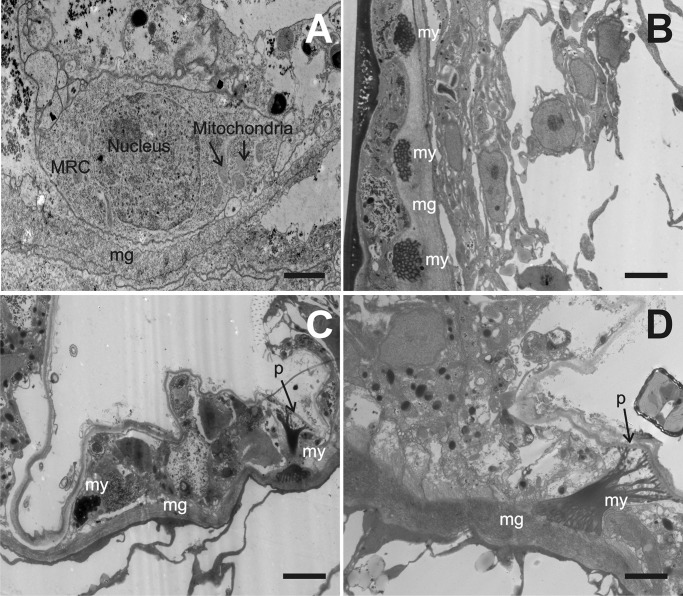
**Transmission electron micrographs of polyp-stolon junctions of fixed colonies of *Podocoryna carnea*.** (A) A mitochondrion-rich cell (MRC) adjacent to the thickened layer of mesoglea (mg) in the polyp-stolon junction. Scale bar: 1 µm. (B) Several bundles of myonemes (my) from myoepithelial cells are shown within the mesoglea. Note the regular spacing. Scale bar: 2 µm. (C) Two bundles of myonemes from myoepithelial cells are visible. The myonemes on the right extend from the mesoglea to the perisarc (p). Scale bar: 2 µm. (D) Myonemes from a myoepithelial cell extending from the perisarc to the mesoglea. Scale bar: 2 µm.

Polyp-stolon junctions of colonies of *P. carnea* were also examined using TEM. As previously, mitochondrion-rich cells adjacent to a thickened layer of mesoglea were observed (Fig. 6A). Bundles of myonemes were observed on the ectodermal side of the thickened mesoglea (Fig. 6B). These myonemes extend from the thickened mesoglea to the rigid perisarc on the outside of the colony (Fig. 6C,D).

### Response of ROS emissions at polyp-stolon junctions to functional manipulations

**Fig. 7.**
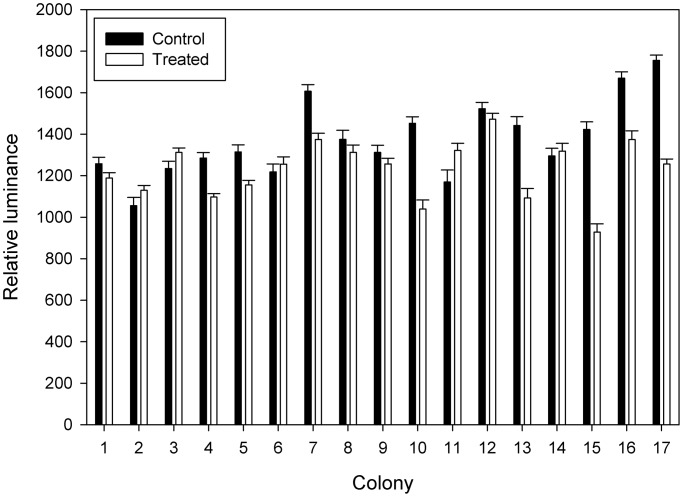
**Levels of reactive oxygen species in mitochondrion-rich cells of colonies of *Podocoryna carnea* as measured by H_2_DCFDA fluorescence.** Mean±s.e.m. relative luminance (foreground minus background fluorescence, gray scale 0–4095) of mitochondrion-rich cells for three polyp-stolon junctions per colony are shown. Colonies treated with 2,4-dinitrophenol (open bars) produce overall significantly less reactive oxygen species than control colonies (filled bars). To ensure rapid measurement of all colonies, experiments were carried out in groups of 3–4 colonies. Colonies are paired in the order they were measured.

Previous investigations of uncouplers (Blackstone, 1998b) focused on the redox state of mitochondrion-rich cells by examining the fluorescence of NAD(P)H. Here, complementary experiments were carried out examining ROS. As measured by relative luminance, control mitochondrion-rich cells overall exhibited greater fluorescence than those treated with DNP (grand mean±s.e.m. 1369.2±9.8 vs 1232.8±8.4). Using colonies within treatment as the error variance in an analysis of variance, relative luminance showed a significant difference between treatments (Fig. 7; *F*=7.2; d.f.=1,32; *P*=0.01). Examination of the coefficients of the variance components suggested that the *F* statistic was slightly conservative. The foreground area of the control mitochondrion-rich cells was also slightly larger than that of the treated (grand mean±s.e.m. 9.1±0.05 and 8.7±0.05 µm^2^) but not significantly so (*F*=2.6; d.f.=1,32; *P*=0.12). Treated colonies exhibited significantly greater background fluorescence than controls (*F*=5.5; d.f.=1,32; *P*=0.03). No difference in the number of mitochondrion-rich cells was found (*F*=0.8; d.f.=1,32; *P*=0.78).

**Fig. 8.**
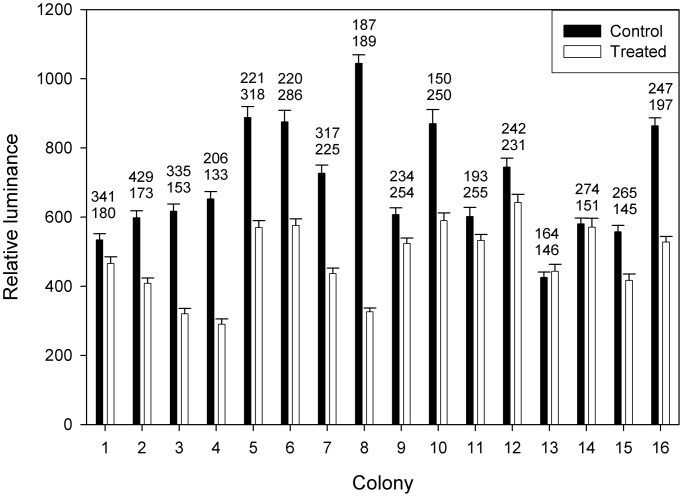
**Mitochondrion-rich cells at polyp-stolon junctions in surgically manipulated colonies of *Podocoryna carnea*.** Half of each of the 16 colonies was removed except for three polyps. The other (control) half was undisturbed. After 1–2 weeks, levels of reactive oxygen species were assayed by H_2_DCFDA fluorescence (mean±s.e.m.) for the three manipulated polyps (unfilled bars) and three comparable control polyps (filled bars). Numbers of mitochondrion-rich cells were also counted for manipulated (lower number) and control (upper number) polyps. To ensure rapid measurement of all colonies, experiments were carried out in groups of 4 colonies.

To further examine the functional biology of mitochondrion-rich cells, an experiment using microsurgery was carried out. Control polyps exhibited more mitochondrion-rich cells (Fig. 8), but this difference was not significant (*N*=16; t=1.76; *P*=0.1). Control polyps also exhibited greater mean relative luminance of mitochondrion-rich cells (Fig. 8), and this difference was highly significant (*N*=16; t=4.87; *P*=0.0002). Eliminating stolons thus diminished the ROS of mitochondrion-rich cells.

## DISCUSSION

Experiments using the fluorescent probes H_2_DCFDA, rhodamine 123, Hoechst 33342, and negative controls support the hypothesis of clusters of mitochondria at polyp-stolon junctions. We interpret these clusters of mitochondria as being part of mitochondrion-rich myoepithelial cells. Confocal microscopy shows that these are large columnar cells extending through much of the polyp-stolon junction.

Mitochondrion-rich cells develop in areas of strong metabolic demand. We suggest that the mitochondrion-rich cells at polyp-stolon junctions operate a complex valve that regulates gastrovascular flow. Our interpretation of the uncoupler experiment is thus straightforward. The uncoupler lowers mitochondrion membrane potential, triggers ATP synthesis, and shifts the redox state of the electron carriers in the direction of oxidation. Less ROS are therefore formed. Since redox state in general and ROS in particular have a signaling function (Warren et al., 2014), these results suggest that part of the function of mitochondrion-rich cells may be related to their redox state and production of ROS (Blackstone, 2001, 2003). Our interpretation of the stolon-removal experiment follows this conceptualization. If a polyp is not attached to the stolons of a colony, the valve is not pulled open. Metabolic demand diminishes and over a period of days, mitochondrion-rich cells regress. ROS emissions from these cells decrease correspondingly. The TEM results illuminate the mechanism by which the mitochondrion-rich cells actually pull open the valve. The myonemes of these cells attach to the mesoglea and to the exterior perisarc. A reasonable explanation for the thickened mesoglea in this area is that the adjacent ectodermal mitochondrion-rich cells are pulling on it and thus pulling open the lumen. Because of this mechanical stress, the mesoglea develops into a much thicker layer than elsewhere in the colony.

After revisiting the studies of hydroid polyp-stolon junctions, a clear hypothesis for the function of mitochondrion-rich cells can now be proposed. As a polyp grows and begins to consume food, it pumps nutrient-rich gastrovascular fluid out through a narrow valve at the polyp-stolon junction. Myoepithelial cells in this region are subject to increasing metabolic demand as they pull open this valve to allow gastrovascular fluid to enter and leave the polyp. These cells are anchored to the perisarc and pull on the mesoglea, which becomes thickened. The structure of the polyp-stolon junction may show other changes as well (Buss et al., 2013). Given the intense metabolic demand, these myoepithelial cells become mitochondrion rich. The high levels of ROS emissions from the mitochondrion-rich cells and the sensitivity of these emissions to the food supply of the colony results in these cells becoming the locus of colony-wide redox signaling (Blackstone, 1998b, 2001, 2003, 2009; Blackstone et al., 2004a,b, 2005; Harmata and Blackstone, 2011).

## MATERIALS AND METHODS

### Study species

Colonies of *P. carnea* and *H. symbiolongicarpus* were grown on 15 mm round cover glass from a few polyps and the connecting tissue of a source colony, and maintained in racks in aquariums at 20.5°C using standard conditions (e.g. Blackstone, 1998b). Colony growth was confined to one side of the coverslip. Colonies were fed brine shrimp three times a week. Experiments were performed on non-feeding days, and conformed to the relevant regulatory standards for animal welfare.

### Elucidation of the structure of polyp-stolon junctions

Several common fluorescent probes were used to illuminate the structure of polyp-stolon junctions. Briefly, colonies of both species were incubated in seawater containing H_2_DCFDA [dissolved in dimethyl sulfoxide (DMSO) 10 µmol l^−1^] for an hour in the dark. Negative controls were similarly incubated in seawater containing DMSO. In other experiments, colonies were incubated in seawater containing rhodamine 123 (10 µg ml^−1^) for 30 min in the dark, and then rinsed three times for 5 min each (Johnson et al., 1980) in seawater. Negative controls were similarly incubated in seawater. To identify and differentiate nuclei from ROS-emitting mitochondria, colonies were incubated in seawater containing H_2_DCFDA (10 µmol l^−1^) and Hoechst 33342 (5 µg ml^−1^) for an hour in the dark. Following incubation, fluorescence was visualized using a Zeiss Axiovert 135 inverted microscope (Carl Zeiss, Jena, Germany). The following excitation/emission wave lengths were used: NAD(P)H and Hoechst 33342, 365/420–490 nm; H_2_DCFDA and rhodamine 123, 450–490/515–565 nm. Hoechst 33342 is visualized with excitement in the ultraviolet and emission in the blue, and thus is confounded with emissions from both NAD(P)H and chitin. At the concentration used the Hoechst signal is much stronger than that of the NAD(P)H and can be distinguished from chitin by its location. Images of polyp-stolon junctions were acquired using a Hamamatsu Orca-100 cooled CCD (Hamamatsu Photonics, Hamamatsu City, Japan) and Image Pro Plus software, ver. 6.3 (Media Cybernetics, Silver Spring, MD, USA). With a region of interest confined to the polyp-stolon junction, co-localization of fluorescent emissions was estimated using Pearson's correlation, r_p_ (see Blackstone et al., 2004a), calculated in Image Pro Plus. Similar procedures were used when obtaining confocal images with a Zeiss LSM 5-Pascal laser scanning microscope. In the three-dimensional reconstruction of the image stack several factors (seawater medium, two cover glass thickness, examination of 30 µm sections) contributed to spherical aberration and stretching of the Z axis. Nevertheless, the three-dimensional reconstruction closely matches examination of individual sections.

For TEM work, colonies were fixed in 2.5% glutaraldehyde for 3 h at 4°C, then rinsed three times for 10 min each in Millonig's phosphate buffer. Colonies were postfixed in 1% osmium tetroxide for 2 h at room temperature, rinsed three times for 10 min each in Millonig's phosphate buffer, and then dehydrated in ethanol series and cleared in acetone. Polyps and stolons were removed and infiltrated and embedded in Embed 812 resin and sectioned using a diamond knife on an ultramicrotome. Thin sections were collected on Formvar-coated slot grids or 75-mesh copper grids. Sections were stained with uranyl acetate and lead citrate. Sections were examined using a Hitachi H-600 transmission electron microscope and digital micrographs were acquired. Mitochondrion-rich cells can be visualized by transverse sections, while myonemes are best visualized using oblique sections.

### Response of ROS emissions at polyp-stolon junctions to functional manipulations

Using standard methods, colonies of *P. carnea* were incubated in 30 µmol l^−1^ 2,4-dinitrophenol in seawater for 48 h. Controls were similarly incubated in seawater. Both treated and control colonies were maintained in the dark except when being fed or when the water was changed. After 48 h of incubation, H_2_DCFDA (10 µmol l^−1^) was added for one additional hour of incubation in the dark, and three polyp-stolon junctions per colony were imaged as described above. For each image, all measurable fluorescent objects were identified, and the gray-scale (0–4095) luminance of the foreground (the putative cell) and background (a roughly equivalent-sized area around the cell) of each of these was determined using Image Pro Plus. To do this, a circular area of interest (21 µm^2^) was placed around an individual fluorescent object and the area and mean luminance of the bright (foreground) and complementary dark (background) region were automatically determined. Relative luminance was calculated by subtracting the background from the foreground. Data were analyzed with an analysis of variance in SAS software.

Removal of the stolons surrounding a polyp-stolon junction should eliminate the need to open the valve. Without the metabolic demand associated with pulling open the valve, over a timescale of days, the number of mitochondria within each cell should diminish as should the number of electron transport chains within each mitochondrion. While ROS can vary depending on metabolic demand and the redox state of the electron carriers, generally more mitochondria and more electron transport chains will produce more ROS. ROS should thus decrease at polyp-stolon junctions that are devoid of stolons. Colonies of *P*. *carnea* were grown as described above. When the colony covered one side of the cover glass, half was left undisturbed while the other half was entirely removed with a micro-scalpel except for three polyps. With care, the stolons attached to these three polyps were also removed. Colonies were maintained in this fashion for between 1 and 2 weeks with new stolon growth removed each day. As described above, colonies were then incubated in H_2_DCFDA and imaged. For each colony, images of the three manipulated polyps and three comparable polyps from the control half were obtained. The numbers of mitochondrion-rich cells were counted, and the relative luminance of each mitochondrion-rich cell was calculated as described above. Results were analyzed using paired-comparison *t*-tests.
